# Post-synthesis of Sn-beta zeolite by aerosol method†

**DOI:** 10.1039/d2ra06366b

**Published:** 2023-02-07

**Authors:** Guang Xiong, Huaxiang Yang, Liping Liu, Jiaxu Liu

**Affiliations:** a State Key Laboratory of Fine Chemicals, School of Chemical Engineering, Dalian University of Technology Dalian 116024 China gxiong@dlut.edu.cn +86-411-84986340

## Abstract

Sn-beta zeolite is a Lewis acid catalyst which can activate the C–O and C=O bonds of many organic compounds. In this paper, a simple aerosol method has been firstly applied to the post-synthesis of Sn-beta zeolite. The aqueous solution containing SnCl_2_ and dealuminated beta zeolite was rapidly dried using an aerosol generator to obtain the Sn-beta zeolites with different Sn contents. The physicochemical properties of the Sn-beta zeolites were further characterized by XRD, nitrogen adsorption–desorption, FT-IR and Py-FT-IR techniques. The catalysts exhibited good catalytic performances in the Baeyer–Villiger oxidation reaction of cyclohexanone.

## Abstract 2

Sn-beta zeolites with different Sn contents can be prepared by an aerosol-assisted post-synthesis method.

## Graphical abstract

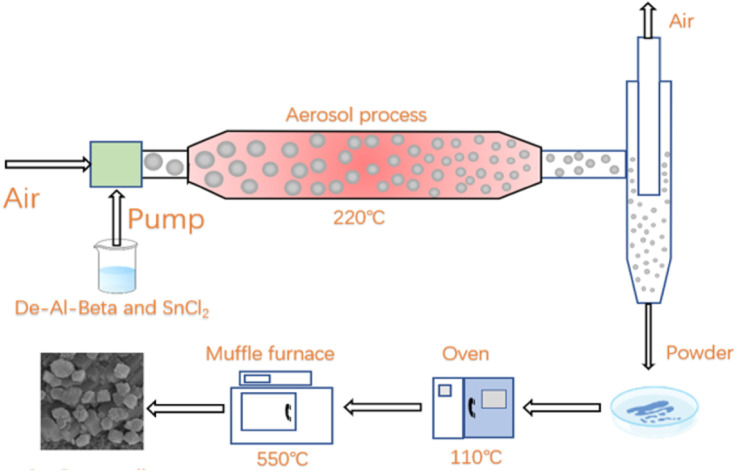

## Introduction

ε-Caprolactone is a very important organic intermediate, and has wide application in the fields of targeted drug delivery, absorbable surgical sutures, artificial implanted tissues, and bioengineering.^1–5^ Methods for the synthesis of ε-caprolactone include a cyclohexanone route and non-cyclohexanone route. The cyclohexanone route refers to the Baeyer–Villiger oxidation reaction of cyclohexanone to produce ε-caprolactone. The oxidants used include *m*-chloroperbenzoic acid, peroxyacetic acid, peroxysuccinic acid, H_2_O_2_, O_2_*etc.*^6–10^ H_2_O_2_ is an atom-economic and environmentally friendly oxidant. The oxidation of cyclohexanone with H_2_O_2_ requires a catalyst containing Lewis acidity. Sn-beta zeolite is considered an ideal catalyst for the Baeyer–Villiger oxidation reaction of cyclohexanone to obtain ε-caprolactone.

Many methods have been used to synthesize the Sn-beta zeolite. In 1997, Mal *et al.*^11^ reported the hydrothermal synthesis of Sn–Al-beta zeolite by adding SnCl_4_·5H_2_O to the silica-alumina gel. After dealumination there are still a small amount of aluminum atoms and silanol nests in the zeolitic framework. Therefore, the Sn-beta zeolite exhibited poor hydrophobicity, which severely affected its catalytic performance. Subsequently, Corma *et al.*^12^ synthesized the Sn-beta zeolite with high hydrophobicity by using HF as the mineralizer. However, the synthetic liquid was close to neutral due to the use of HF, resulting in a long crystallization time and large crystal size. The synthesis of nano- and hierarchical Sn-beta zeolites is an effective way to reduce the diffusion resistance.^13–15^ Wu *et al.*^16^ used *N*-cyclohexyl-*N*,*N*-dimethylcyclohexanaminium hydroxide as a structure-directing agent to hydrothermally synthesize nano Sn-beta zeolite under alkaline conditions. Parulkar *et al.*^17^ hydrothermally synthesized Sn-beta zeolite with a size of less than 200 nm in a fluorine-free medium by using 4,4′-trimethylenebis (TMP) as a structure-directing agent. Although the size of zeolite has been reduced, the hydrophobicity of the Sn-beta zeolite is not satisfied. Tang *et al.*^18^ used polydiallydimethylammonium chloride (PDADMAC) and TEAOH as templates to synthesize hydrophobic hierarchical Sn-beta in F^−^ containing medium. The Sn-beta zeolite exhibited good activity and reusability in the conversion of cellulosic sugar to methyl lactate.

Post-synthesis of Sn-beta zeolite means that the Sn species is implanted into the framework by occupying the ‘T’ vacancy. The post-synthesis method can be divided into three types: gas–solid, liquid–solid and solid–solid ion exchange. Li *et al.*^19^ and Liu *et al.*^20^ reported the synthesis of the Sn-beta zeolite by introducing the SnCl_4_ vapor into the dealuminized zeolite at high temperature (400–500 °C). Tang *et al.*^21^ Grinded the mixture of (CH_3_)_2_SnCl_2_ and the dealuminated beta zeolite. Then the powder was calcined at 550 °C for 6 h to obtain Sn-beta zeolite. The Sn-beta zeolite achieves very high catalytic activity and selectivity for 1,2-diol in the ring-opening hydration of cyclohexene oxide. Dijkmans *et al.*^22–25^ dissolved SnCl_4_·5H_2_O in the isopropanol solution, then mixed the solution with the dealuminated beta zeolite. The Sn-beta zeolite was obtained after stirring for 7 h under reflux condition. By using this method almost all Sn species are implanted into the framework when the Sn loading is below 2 wt%. In summary, the Sn-beta zeolite synthesized by the hydrothermal method has the advantages of high crystallinity and strong hydrophobicity. However, the zeolite crystal size is large and the toxic F^−^ has to be used as a mineralizer. The Sn-beta zeolite prepared by the post-synthesis method has the advantages of small size and high Sn content. However, hydrophobicity of the Sn-beta zeolite is poor because the Sn species cannot completely occupy the silanol nests generated by dealumination.

Aerosol technology is often used to synthesize mesoporous and macroporous materials. Our group previously reported the synthesis of TS-1, Sn-beta, beta and ZSM-5 zeolites by the aerosol-assisted hydrothermal method.^26–30^ It was found that the aerosol technique improved the heteroatom dispersion in the precursor and the zeolite. In particular, the microporous and hierarchical Sn-beta zeolite was successfully synthesized with the aid of F^−^ by the same method.^31,32^ The Sn-beta zeolite synthesized by the aerosol-assisted hydrothermal method has the advantages of short crystallization time and less amount of template. The obtained Sn-beta zeolite exhibited excellent catalytic performance in the Baeyer–Villiger oxidation reaction of cyclohexanone. However, the use of toxic F^−^ as a mineralizer prevents its application in large scale production. Therefore, the aim of this paper is to develop a new post-synthesis method without the use of F^−^. Inspired by the advantages of fast drying and good heteroatom dispersion, the aerosol method was firstly applied to the post-synthesis of Sn-beta zeolite in this study. The obtained zeolites were characterized by various techniques. The catalytic performance of the Sn-beta zeolite was tested by the Baeyer–Villiger oxidation reaction of cyclohexanone.

## Experimental section

### Materials

All chemicals are purchased directly from the factory without further purification and processing. The parent Al-beta zeolite with Si/Al = 11 was purchased from Shandong Qilu Huaxin Industrial Co., Ltd. Other chemicals include HNO_3_ (68 wt%, Tianjin Damao Chemical Reagent Factory), 1,4-dioxane (Tianjin Jindong Tianzheng Fine Chemical Reagent Factory), cyclohexanone (Tianjin Fuyu Fine Chemical Co., Ltd), H_2_O_2_ (Tianjin Fuyu Fine Chemical Co., Ltd), chlorobenzene (Sinopharm Chemical Reagent Co., Ltd), SnCl_2_·2H_2_O (Tianjin Damao Chemical Reagent Factory) and chlorobenzene (Sinopharm Chemical Reagent Co., Ltd).

### Synthesis of Sn-beta zeolite

**Scheme 1:**
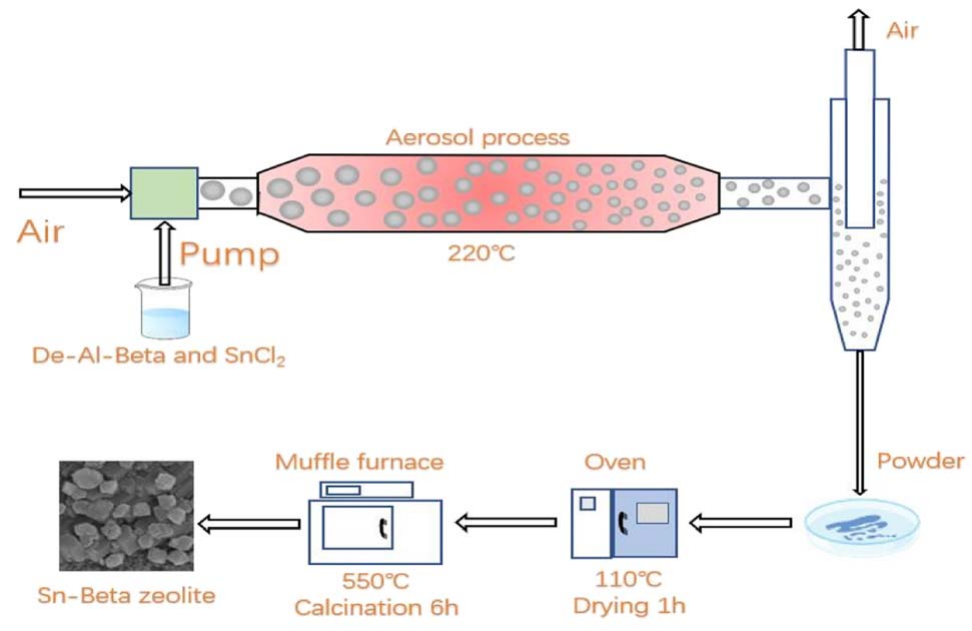
A typical synthesis process of the Sn-beta zeolite by aerosol method.

The parent Al-beta zeolite was dealuminated with nitric acid (68 wt%, 10 mL g^−1^ zeolite) at 85 °C for 24 h. The beta zeolite after dealumination (De-Al-beta) was filtered and washed until the filtrate was nearly neutral. Then the De-Al-beta zeolite was dried at 110 °C overnight and calcined at 550 °C for 6 h. The preparation of the Sn-beta zeolite was carried out by means of solid–liquid ion exchange. A typical synthesis process of the Sn-beta zeolite by aerosol method is shown in Scheme 1.

2 g De-Al-beta was mixed with different mass fractions of SnCl_2_·2H_2_O aqueous solution. After stirring for 5 h, the aerosol generator was used for rapid spray drying. The obtained powder was further dried at 110 °C for 1 h to remove the small amount of the remaining water. Finally, the powder was calcined at 550 °C for 6 h in the air. The products were denoted as Sn-beta-*x*, where *x* is the weight percent of Sn atom in the Sn-beta zeolite.

### Characterization

X-ray diffraction (XRD) patterns of the zeolites were recorded on a Rigaku Smart Lab 9 kW diffractometer by using nickel-filtered CuKα X-ray source. The experiment was operated at 40 kV and 100 mA with a range of 2 theta = 5–50° and at a scanning rate of 10° min^−1^. The elemental composition of the Sn-beta zeolite was analyzed by induced coupled plasma-atomic emission spectroscopy (ICP-AES) on PerkinElmer/AVIO 500.

The adsorption/desorption isotherms were measured with JingWeiGaoBo JW-TB440A instrument using N_2_ as an adsorbate at 77 K. The samples were heat-treated at 350 °C for 1 h under vacuum prior to the measurement. Total surface area was calculated on the basis of Brunauer–Emmett–Teller (BET) method. The micropore volume and micropore area were calculated *via* the *t*-plot method. Ultraviolet-visible diffuse reflectance spectra (UV-vis) were recorded with Hitachi U-4100 in the range of 190–600 nm by using BaSO_4_ as the reference. The acid properties of the catalyst were measured by FT-IR spectra with pyridine adsorption on Thermo Nicolet IS50. First, the samples were pretreated at 400 °C for 1 h under vacuum. FT-IR spectrum of the –OH region was collected with spectral resolution of 4 cm^−1^ after cooling down to room temperature, which was also used as the background for pyridine adsorption studies. Then the pyridine adsorption was performed at room temperature for 30 min and desorb under vacuum at desired temperature for 35 min. The pyridine-FT-IR spectra were then collected.

### Catalytic tests

The catalytic performances of the Sn-beta zeolites were evaluated by the Baeyer–Villiger oxidation of cyclohexanone with hydrogen peroxide (30 wt%). The reaction was carried out in a 100 mL round bottom flask equipped with condensed water under stirring. The reaction was carried out as follows: 50 mg of the catalyst was added to a 100 mL round bottom flask, then 7.65 mL of 1,4-dioxane (solvent), 2 mmol of cyclohexanone, 2 mmol of H_2_O_2_ (30 wt%), and 2 mmol of chlorobenzene (internal standard) were added in turn. The mixture was quickly transferred to a water bath at 90 °C and treated for 2 or 3 hours. After the reaction is completed, the mixture is rapidly cooled to room temperature. After centrifugal separation, the upper reaction solution was analyzed by gas chromatography with FULI-9790 hydrogen flame ionization detector. The chromatographic column is SE-54 (30 m × 0.32 mm × 0.5 YM), the column temperature is 80 °C, the detector temperature is 200 °C, the injector temperature is 200 °C. The internal standard method was used for quantification. GC-MS (Agilent-5975C) was used to identify the products after the reaction. The main product is ε-caprolactone and the main by-product is 6-hydroxycaproic acid. The conversion of cyclohexanone, the selectivity of ε-caprolactone, and the yield were calculated from the following equations:
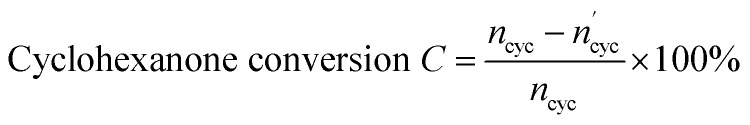

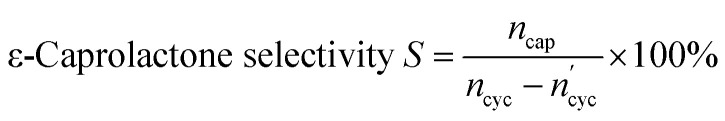
ε-Caprolactone yield *Y* = *C* × *S* × 100% Here, *n*_cyc_ represents the amount of the cyclohexanone before the reaction, and 
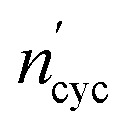
 represents the amount of the cyclohexanone remaining after the end of the reaction. *n*_cap_ is the amount of the produced ε-caprolactone species.

## Results and discussion

**Fig. 1:**
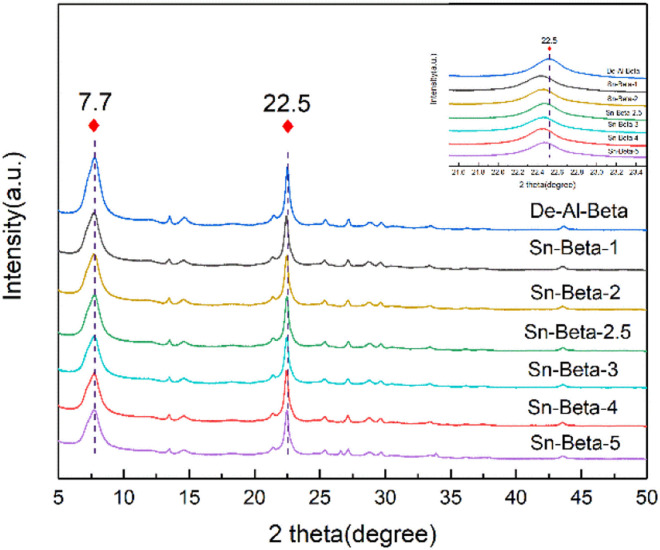
XRD patterns of De-Al-beta and Sn-beta zeolites with different Sn contents.

After the parent Al-beta zeolite was deeply dealuminated with concentrated HNO_3_, the Si/Al increased from 11 to more than 1700 (Table 1). This indicates that most of the Al species in the zeolite were removed. Fig. 1 shows the XRD patterns of the De-Al-beta and Sn-beta zeolites with different Sn contents. It can be seen that all the samples show the characteristic peaks of beta zeolite. As shown from the inset in Fig. 1, the *d*_302_ spacing of the BEA* matrix expands from 3.9483 Å (De-Al-beta, 2*θ* = 22.5°) to 3.9605 Å (Sn-beta, 2*θ* = 22.43°), indicating that the Sn species has been successfully implanted into the zeolite framework. Table 1 shows the relative crystallinities of all the samples. The relative crystallinities decrease as compared to that of De-Al-beta, which may be caused by HCl generated by partial hydrolysis of SnCl_2_·2H_2_O.^33^ In addition, it has been reported that the framework Sn will be partially hydrolyzed to form open Sn sites.^34,35^

**Table 1:** Textural properties of De-Al-beta and Sn-beta with different Sn contents

Sample	*S*_BET_ a (m^2^ g^−1^)	*S*_ext_ b (m^2^ g^−1^)	*V*_total_ c (cm^3^ g^−1^)	*V*_micro_ d (cm^3^ g^−1^)	*V*_meso_ e (cm^3^ g^−1^)	RC^f^ (%)
De-Al-beta	686	145	0.48	0.22	0.26	100
Sn-beta-1	668	144	0.49	0.21	0.28	78
Sn-beta-2	651	143	0.45	0.20	0.25	72
Sn-beta-2.5	638	138	0.46	0.20	0.26	78
Sn-beta-3	623	140	0.45	0.19	0.26	68
Sn-beta-4	633	137	0.46	0.20	0.26	78
Sn-beta-5	619	132	0.42	0.19	0.23	66

a BET surface area.

b External surface area.

c *P*/*P*_0_ = 0.99.

d *t*-plot method.

e *V*_total_ − *V*_micro_.

f Relative crystallinity, calculated from the sum of the 2*θ* = 7.6° and 2*θ* = 22.5° peak areas.

**Fig. 2:**
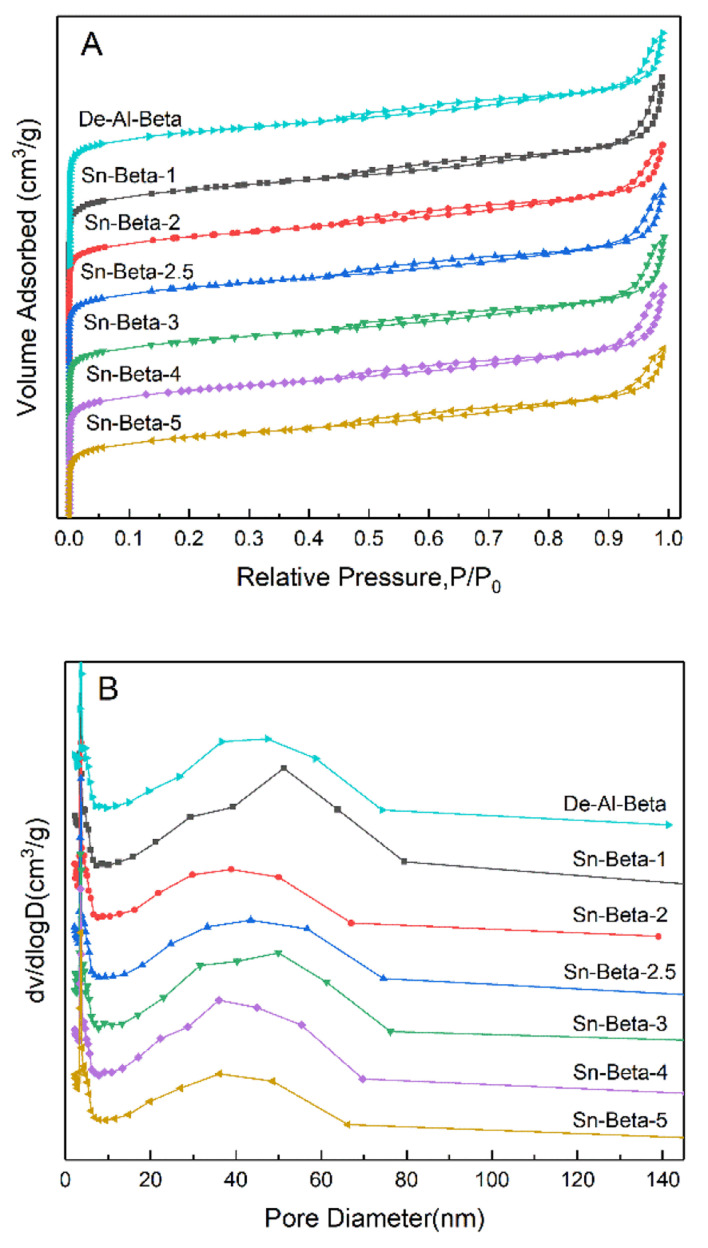
Nitrogen adsorption–desorption isotherms (A) and corresponding pore size distributions (B) of De-Al-beta and Sn-beta zeolites with different Sn contents.

The N_2_-adsorption–desorption isotherms of the De-Al-beta and Sn-beta zeolites with different Sn contents are shown in the Fig. 2(A). All the samples show the intermediate typeI–type IV isotherms. At low pressure, the isotherms show a sharp upward trend, indicating the presence of the micropores. The appearance of the hysteresis loops suggests that both the dealuminated beta and the Sn-beta zeolites contain the intercrystalline mesopores. The Fig. 2(B) shows the pore size distribution in the range of 10–70 nm. It can be seen from the Table 1 that the specific surface areas and the pore volumes of the samples tend to decrease slightly with the increase of the Sn content. The decrease trend is more obvious at the higher Sn loading, which is probably due to the formation of SnO_2_ particles.

**Fig. 3:**
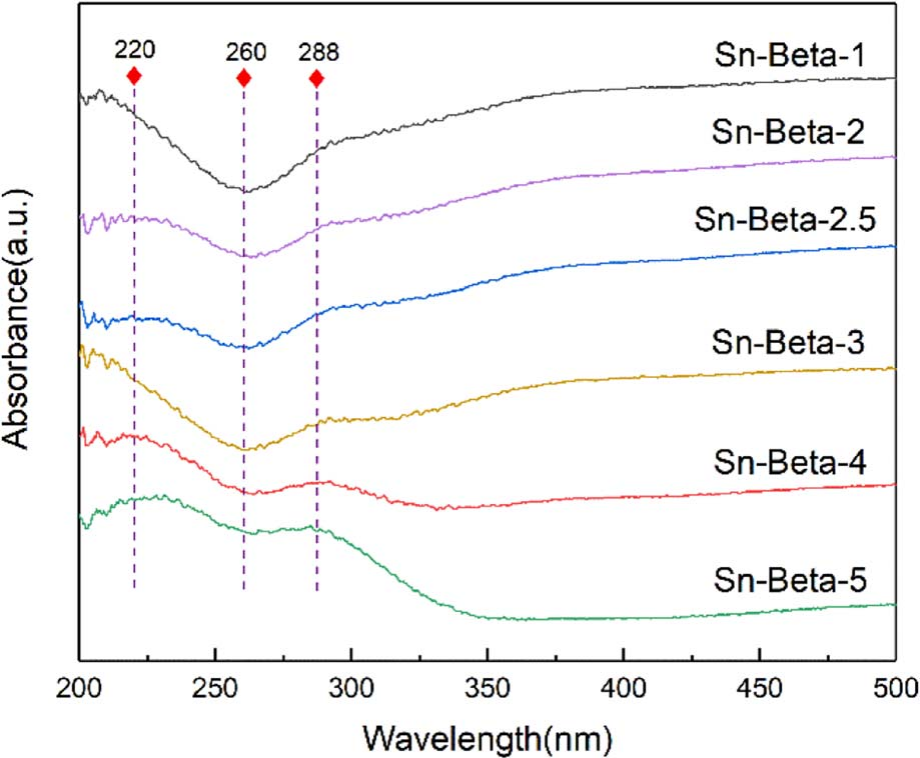
UV-vis spectra of the Sn-beta zeolites with different Sn contents.

Fig. 3 shows the UV-Vis spectra of the Sn-beta zeolites with different Sn contents. All the samples show the absorption peaks at 220 nm, indicating that Sn^4+^ has been successfully implanted into the hydroxyl nests in tetra-coordination.^36,37^ In addition, the samples do not exhibit the peak at around 260 nm, indicating the absence of six-coordinated hydrated forms of Sn species or SnO_*x*_ clusters.^25,38^ An absorption peak at 288 nm is assigned to the formation of the SnO_2_ particles.^39,40^ The peak is more obvious when the loading exceeds 3 wt%. It can be concluded that the tetrahedral framework Sn is the dominate Sn species at low loading, while both the tetrahedral framework Sn and the SnO_2_ particles are obviously present at high loadings.

**Fig. 4:**
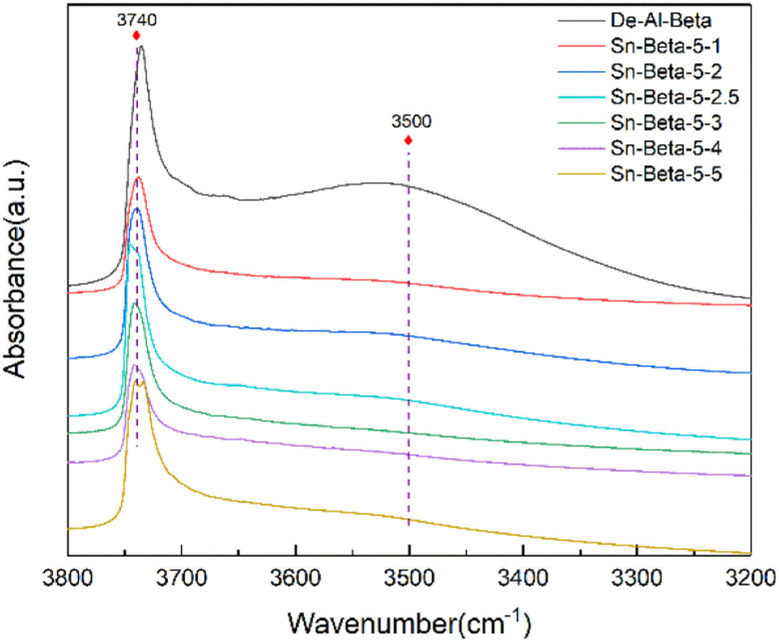
FT-IR spectra of De-Al-beta and the Sn-beta with different Sn contents.

In order to explore the interaction between the Sn species and the hydroxyl nests in the zeolite, the stretching vibration of the hydroxyl groups were observed by FT-IR spectroscopy. As shown in Fig. 4, all the samples show the absorption peaks at 3740 cm^−1^, indicating the existence of the surface silanol groups or the terminal silanol.^41,42^ The peaks at around 3500 cm^−1^ are attributed to the stretching vibrations of Si–OH in the silanol nest of the zeolite.^41^ The De-Al-beta zeolite shows a broad absorption band in the range of 3400–3600 cm^−1^ with a maximum at 3500 cm^−1^, which is typical in the literature.^43^ This broad absorption band was assigned to bridged hydroxyl groups which are perturbed by H-bond interactions,^44^ indicating that a large number of the silanol nests were produced due to the dealumination. After the introduction of the Sn species the absorption peaks at around 3500 cm^−1^ disappear, indicating that the Sn^2+^ species has successfully entered the ‘T’ vacancy.

**Fig. 5:**
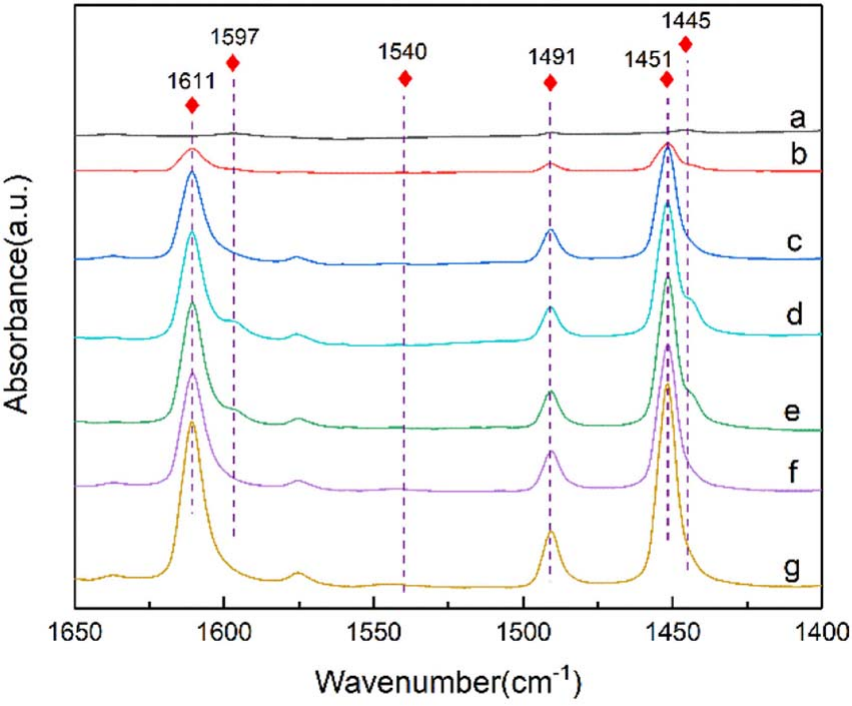
FT-IR spectra of the De-Al-beta (a), Sn-beta-1 (b), Sn-beta-2 (c), Sn-beta-2.5 (d), Sn-beta-3 (e), Sn-beta-4 (f) and Sn-beta-5 (g) samples after pyridine adsorption at 298 K for 30 min and desorption at 423 K for 35 min.

Infrared spectroscopy using pyridine as probe molecules can explore the acid properties of the zeolite. The dealumination and Sn incorporation remove the Brønsted acid sites and introduces the Lewis acid sites, which are the active centers of the B–V oxidation of cyclohexanone (Fig. S1†). Fig. 5 shows the pyridine adsorption infrared spectra of the De-Al-beta and Sn-beta zeolites. The absorption peaks of the De-Al-beta sample at 1445 cm^−1^ and 1597 cm^−1^ are attributed to hydrogen-bonded pyridine molecules.^45^ There is no characteristic peak assigned to Brønsted acid at 1540 cm^−1^.^46,47^ After implanting Sn^4+^ into the framework, the characteristic peaks at 1611 cm^−1^ and 1451 cm^−1^ are present. These peaks represent the Lewis acid sites.^45,48^ The peak intensities tend to increase with increasing the Sn content. The peak at 1491 cm^−1^ is the overlap of the Brønsted and Lewis acid sites. Due to the absence of the Brønsted acid sites the peak should be assigned to the Lewis acid sites in this study. Table 2 shows the Si/Sn molar ratio of the Sn-beta zeolites with different Sn contents. The Si/Sn ratio shows a decreasing trend with the increase of Sn content. When the Sn content exceeds 4 wt%, the Si/Sn molar ratio show a slight decrease. According to literature,^49^ the Lewis acid content in Sn-beta zeolite can be calculated using the peak area at 1451 cm^−1^. As shown in Table 2 the amount of the Lewis acid sites increases with the decrease of Si/Sn molar ratio. This indicates that the introduced Sn species created the Lewis acid sites. The Lewis acid content in the Sn-beta-5 increases to a lesser extent, which is in accordance with the trend of Si/Sn molar ratio. This indicates that the loss of the Sn species occurred when the Sn loading is higher.

**Table 2:** Si/Sn molar ratios and Lewis acid content of Sn-beta zeolites with different Sn contents

Sample	Si/Sn^a^	LS^b^ (μmol g^−1^)
De-Al-beta	∞	0
Sn-beta-1	165	63
Sn-beta-2	42	157
Sn-beta-2.5	37	171
Sn-beta-3	32	172
Sn-beta-4	24	216
Sn-beta-5	22	218

a Determined by ICP-OES.

b Calculating according to the equation given in the literature.^49^

**Fig. 6:**
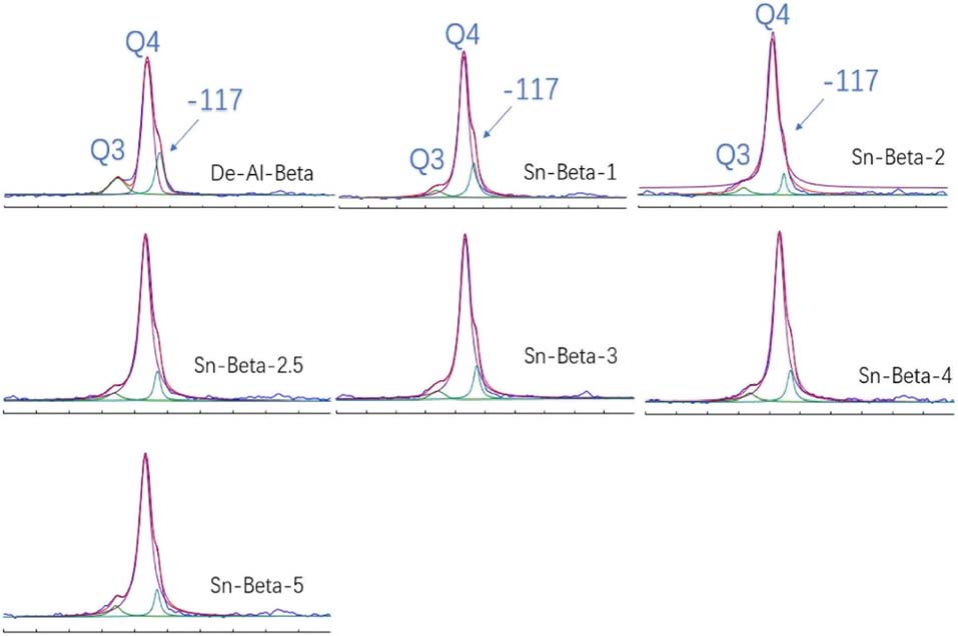
^29^Si MAS NMR spectra of De-Al-beta and the Sn-beta with different Sn contents.

^29^Si MAS NMR spectra is used to detect the environment of Si atom in zeolite, as shown in the Fig. 6. The shoulder peak centers at about −117.2 ppm is commonly attributed to the existence of crystallographically inequivalent sites in the zeolite.^50^ The peaks at −113 ppm and −104 ppm are attributed to Si(OSi)_4_ (*Q*^4^) species and (OSi)_3_Si(OH) (*Q*^3^) species,^51,52^ respectively. It can be seen that the intensity of the peak at −104 ppm (De-Al-beta) is relatively strong, which is caused by the hydroxyl nests generated after the dealumination. After the introduction of Sn, the intensity of the peak at −113 ppm (Sn-beta) decreases, indicating that Sn has successfully occupied the silanol nest.^19^ The values of *Q*^3^/(*Q*^3^ + *Q*^4^) are shown in Table 3. It can be seen that the values of the Sn-beta zeolites are lower than that of the De-Al-beta, indicating that the number of silanol nest in the zeolite decreases. This further indicates that Sn has successfully occupied the silanol nest to form the framework Sn. However, when the Sn content exceeds 3%, the value tends to rise, which is not consistent with the Lewis content. We speculate that the high concentration of Sn precursor in the synthesis process may lead to partial hydrolysis of Sn, forming extra-framework Sn, and some of the SnO_*x*_ generated has Lewis acidity,^53^ which results in higher Lewis acid content for Sn-beta-4 and Sn-beta-5.

**Table 3:** The values of *Q*^3^/(*Q*^3^ + *Q*^4^)

Sample	*Q* ^3^/(*Q*^3^ + *Q*^4^) (%)
De-Al-beta	13.9
Sn-beta-1	6.2
Sn-beta-2	4.93
Sn-beta-2.5	4.2
Sn-beta-3	3.96
Sn-beta-4	4.63
Sn-beta-5	5.35

The catalytic performances of the catalysts were evaluated by the Baeyer–Villiger oxidation reaction of cyclohexanone and H_2_O_2_. The main by-product of this reaction is 6-hydroxycaproic acid, which is produced by the hydrolysis of ε-caprolactone. As shown in Table 4, the conversion of cyclohexanone increases when the Si/Sn molar ratio decreases from 165 to 32. The conversion of cyclohexanone on the Sn-beta-3 reaches a maximum of 50.2%. This is due to the increase of the amount of the Lewis acid sites with increasing the Sn content. Further increasing the Sn content, the conversion firstly remains stable, then tends to decrease. Low activity at higher Sn content has also been reported in other literature,^25,55–57^ which may be caused by the formation of extra framework Sn species, which have a proven deleterious effect on the catalytic performances of Sn-beta zeolite.^39,58,59^ In addition, it has also been reported that not all Sn species are active in the reaction. This may be due to the different activities of open and closed Sn frame sites.^34^

**Table 4:** Catalytic performance of the Sn-beta zeolites with different Sn contents in Baeyer–Villiger oxidation reaction of cyclohexanone

Sample	Conversion^a^ (%)	Selectivity (%)	Yield (%)
Sn-beta-1	23.1	60.8	14.1
Sn-beta-2	45.3	59.5	27.0
Sn-beta-2.5	48.7	63.1	30.7
Sn-beta-3	50.2	60.4	30.3
Sn-beta-4	49.7	60.4	30.0
Sn-beta-5	41.8	57.9	24.2

a Reaction conditions: catalyst, 50 mg; cyclohexanone, 2 mmol; H_2_O_2_(30%), 2 mmol; 1,4-dioxane, 7.65 mL; temperature 363 K; time, 2 h.

For all the samples the selectivities to caprolactone are around 60%. The Lewis acid sites in the Sn-beta zeolites can catalyze not only the Baeyer–Villiger oxidation reaction of cyclohexanone, but also the hydrolysis of ε-caprolactone. Therefore, as the conversion increases more ε-caprolactone was formed. At the same time the probability of hydrolysis of ε-caprolactone is also increased. The Sn-beta-2.5, Sn-beta-3 and Sn-beta-4 show the similar yields of ε-caprolactone, which are higher than those of the other samples. Among them the Sn-beta-2.5 was chosen for the following reaction, since the sample contains less Sn content and shows the highest selectivity.

**Scheme 2:**
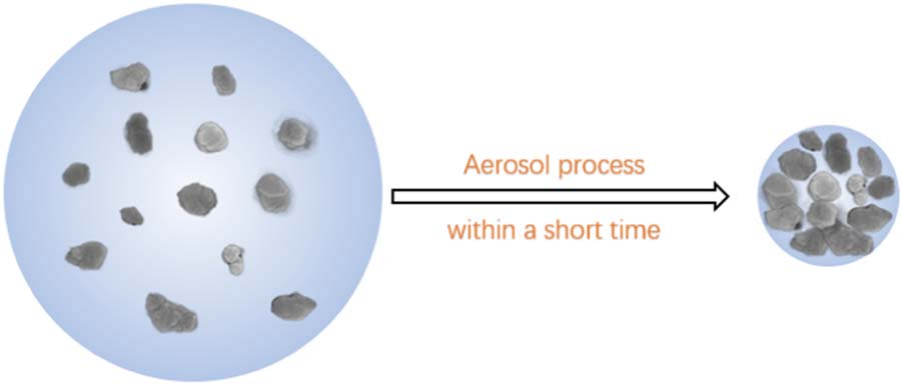
Aerosol technology drying process.

To compare the effect of the different drying process, the Sn-beta-2.5-D zeolite which was prepared *via* the direct drying in the oven was used as a reference. The reaction performances of the Sn-beta-2.5 and Sn-beta-2.5-D were tested by the Baeyer–Villiger oxidation reaction of cyclohexanone. In order to obtain the best caprolactone yield, the reaction time was extended from 2 h to 3 h (FS. 9) It can be seen from the Table 5 that the Sn-beta-2.5 shows both higher conversion of cyclohexanone and higher selectivity to ε-caprolactone, which is consistent with the higher Lewis acid content in Sn-beta-2.5 (TS.2). The use of the aerosol spray drying method reduces the aggregation of Sn species and forms more framework Sn species, which can be verified by the UV-Vis (FS.6) and Py-FT-IR (FS.8) spectra. The mixture of De-Al-beta and the Sn precursor produces the droplets by the aerosol technique (Scheme 2). When the generated droplet passes through the drying zone the water evaporates rapidly. This may create the driving force for the diffusion of the Sn species to the silicon hydroxyl nests inside the zeolite. When the amount of the Sn precursor is high, the decrease of water content in the droplets will also lead to the aggregation of the Sn species. However, oven drying requires longer drying time, which promotes the possibility of aggregation to form the extra-framework Sn species. This will reduce the activity of the catalyst.

**Table 5:** Catalysis performances of Sn-beta-2.5 and Sn-beta-2.5-D in Baeyer–Villiger oxidation reaction of cyclohexanone

Sample	Conversion^a^ (%)	Selectivity (%)	Yield (%)
Sn-beta-2.5	54.3	61.9	33.6
Sn-beta-2.5-D	47.7	57.4	27.4

a Reaction conditions: catalyst, 50 mg; cyclohexanone, 2 mmol; H_2_O_2_(30%), 2 mmol; 1,4-dioxane, 7.65 mL; temperature, 363 K; time, 3 h.

The catalytic performance of the Sn-beta-2.5 catalyst is compared with those reported conducted under the same reaction conditions in the literature. The results are listed in Table 6. Under the same reaction conditions, both the conversion and selectivity in this work are higher than those of the other catalysts. In general, the catalysts prepared by this method show good catalytic performance in the B–V oxidation of cyclohexanone.

**Table 6:** Comparison of the catalysts prepared by different methods

Entry	H_2_O_2_ (wt%)	Conditions	Conversion (%)	Selectivity (%)	References
1	30	3 h, 90 °C	34.5	49.4	54
2	30	3 h, 90 °C	27.4	60.5	55
3	30	3 h, 90 °C	48.7	63.1	This work

**Fig. 7:**
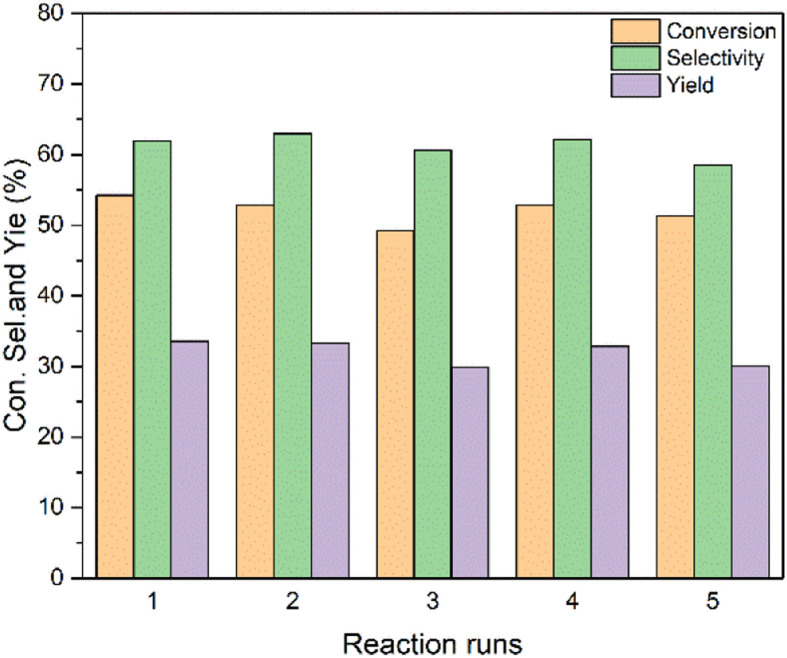
The reusability of the Sn-beta-2.5 catalyst. Reaction conditions: catalyst, 50 mg; cyclohexanone, 2 mmol; H_2_O_2_ (30%), 2 mmol; 1,4-dioxane, 7.65 mL; temperature 363 K; time, 3 h. The main byproduct was 6-hydroxycaproic acid.

Reusability is an important indicator for catalyst evaluation. The Sn-beta-2.5 was chosen to explore the reusability of catalyst. After each reaction, the catalyst was washed four times with ethanol and then four times with deionized water, dried at 110 °C for 4 h. The catalyst was finally calcined at 550 °C for 4 h in the air. The reaction results are shown in the Fig. 7. After repeating 5 times the activity and the selectivity of the catalyst does not show an obvious decrease. The yield of the catalyst decreases from 33.6% to 30.1% after the 5^th^ run, indicating that the Sn-beta zeolite obtained by aerosol drying method has a good reuse performance.

## Conclusions

Sn-beta zeolite was prepared by a novel post-synthesis method. The aerosol-assisted drying technique is used to incorporate the Sn species into the vacancies of the beta zeolite. As compared to the oven drying process the new method is simple, fast and efficient for the dispersion of the Sn species. At the optimal Sn content 2.5 wt%, the catalyst exhibits the conversion of 48.7%, the selectivity of 63.1% and the yield of 30.7% in Baeyer–Villiger oxidation reaction of cyclohexanone. This study provides an alternative route to prepare various heteroatom substituted zeolites.

## Author contributions

Huaxiang Yang: investigation, data curation, original drift. Jiaxu Liu: characterization (FT-IR), characterization (N_2_-adsorption/desorption). Guang Xiong: conceptualization, methodology, formal analysis, review & editing.

## Conflicts of interest

There are no conflicts to declare.

## Supplementary Material

RA-013-D2RA06366B-s001
