# Supplementary material for: Post-synthesis of Sn-beta zeolite by aerosol method
Source: RSC Adv. 2023 Feb 7;13(7):4835–42. doi: 10.1039/d2ra06366b (PMC9903300; doi:10.1039/d2ra06366b)
Supplement: RA-013-D2RA06366B-s001 [file RA-013-D2RA06366B-s001.pdf]

Electronic Supplementary Material (ESI) for RSC Advances.
This journal is © The Royal Society of Chemistry 2023

Support material

Post-Synthesis of Sn-Beta zeolite by Aerosol method

The synthesis of Sn-Beta-x-D: 0.10g $SnCl_2 \cdot 2H_2O$ was dissolved in 38g deionized water, and 2g De-Al-Beta zeolite was added. After stirring for 5 hours, it was dried in an oven at 110 °C for 3 hours. Finally the powder is calcined at 550 °C for 6 hours to obtain Sn-Beta zeolite, which is denoted as Sn-Beta-x-D.

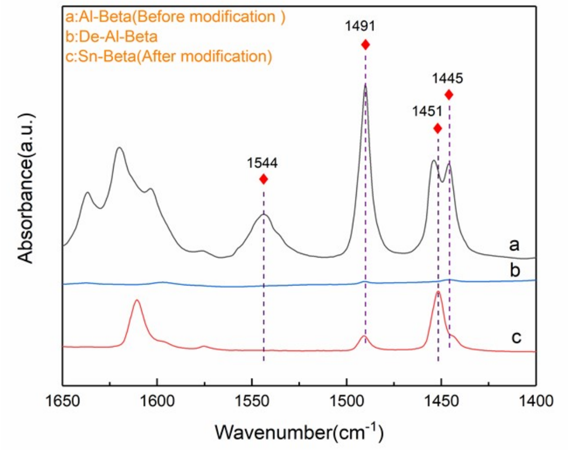

Fig. 1 FT-IR spectra of the Al-Beta, De-Al-Beta and Sn-Beta samples after pyridine adsorption at 298K for 30 min and desorption at 423K for 35 min

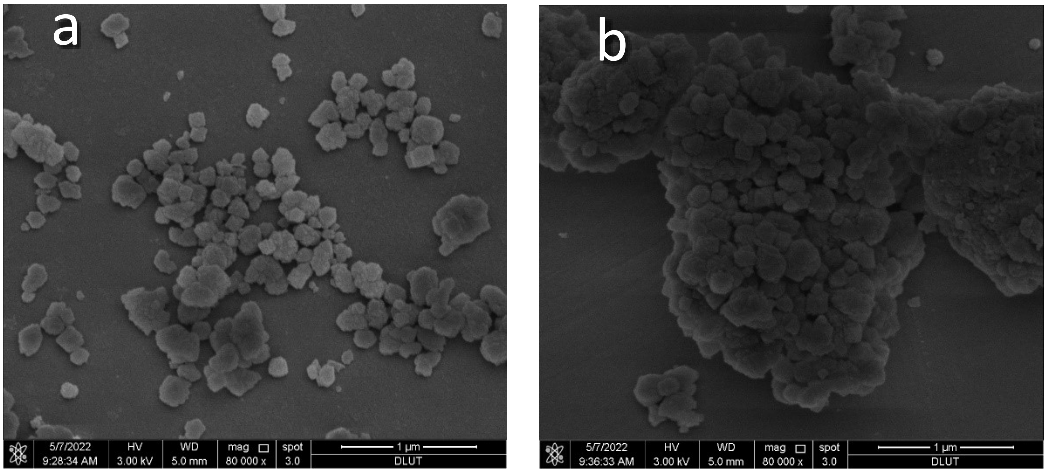

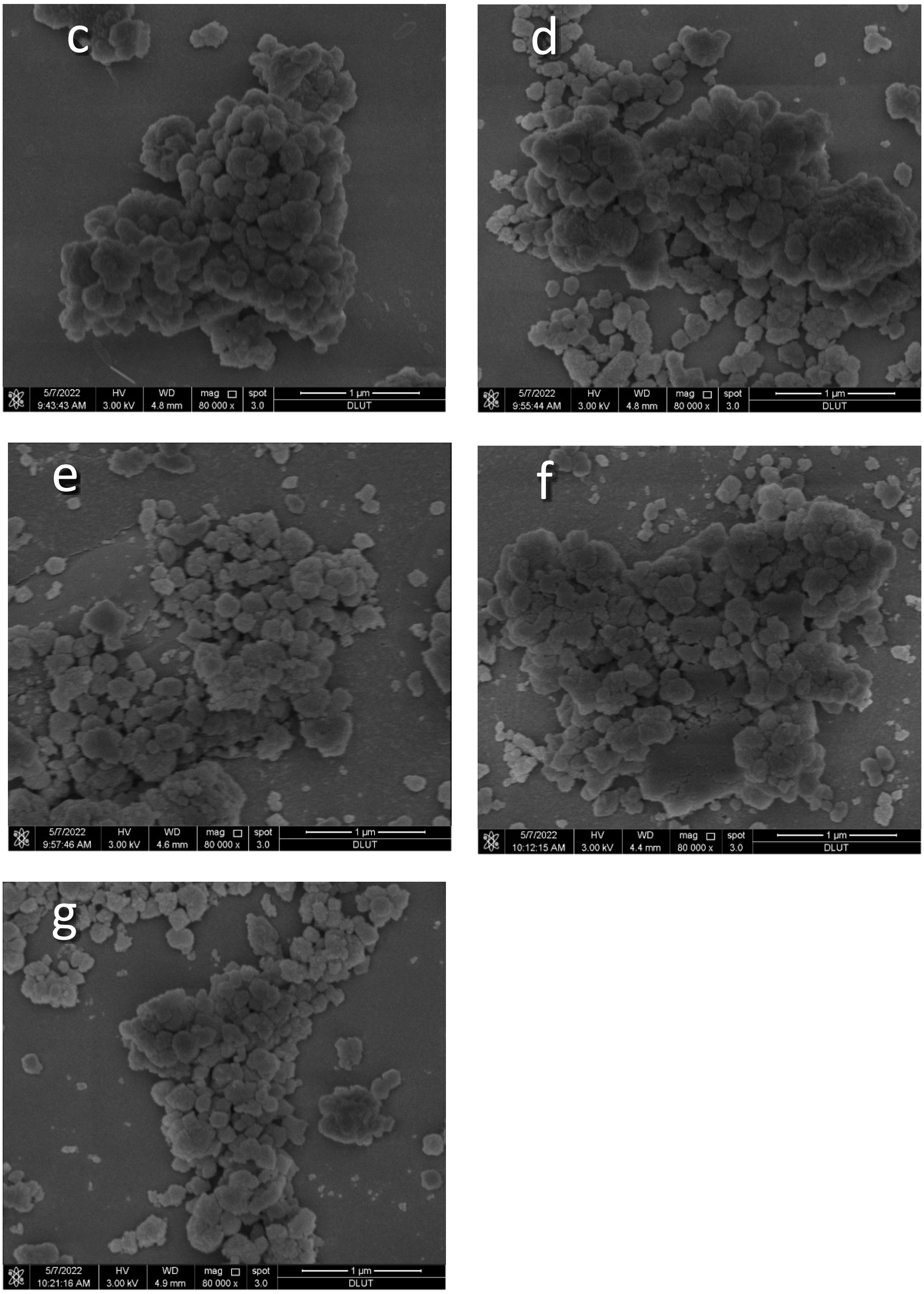

Fig. 2 SEM images of the De-Al-Beta (a), Sn-Beta-1 (b), Sn-Beta-2 (c), Sn-Beta-2.5(d) , Sn-Beta-3(e), Sn-Beta-4(f) and Sn-Beta-5(g) samples

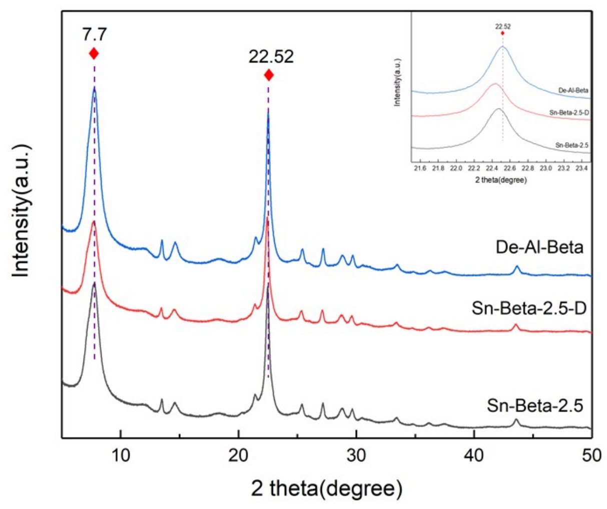

Fig. 3 XRD patterns of De-Al-Beta、Sn-Beta-2.5 and Sn-Beta-2.5-D

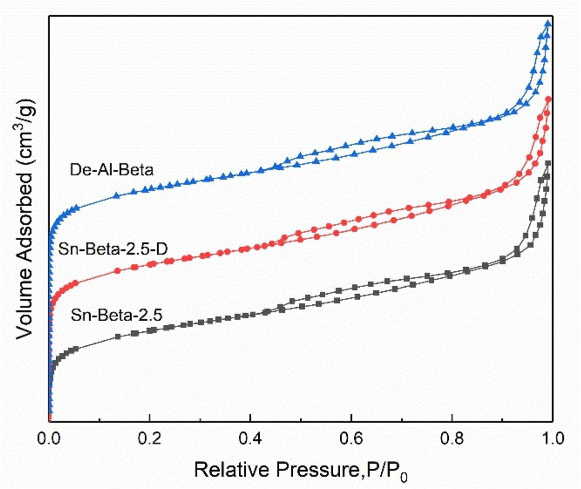

Fig. 4 Nitrogen adsorption-desorption isotherms of De-Al-Beta、Sn-Beta-2.5 and Sn-Beta-2.5-D

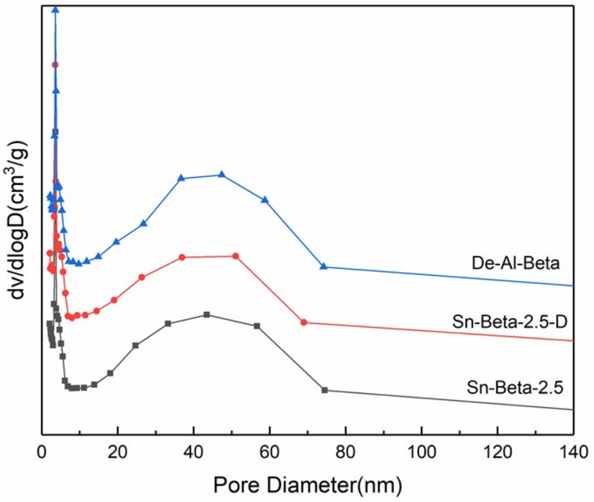

Fig. 5 Corresponding pore size distributions of De-Al-Beta、Sn-Beta-2.5 and Sn-Beta-2.5-D

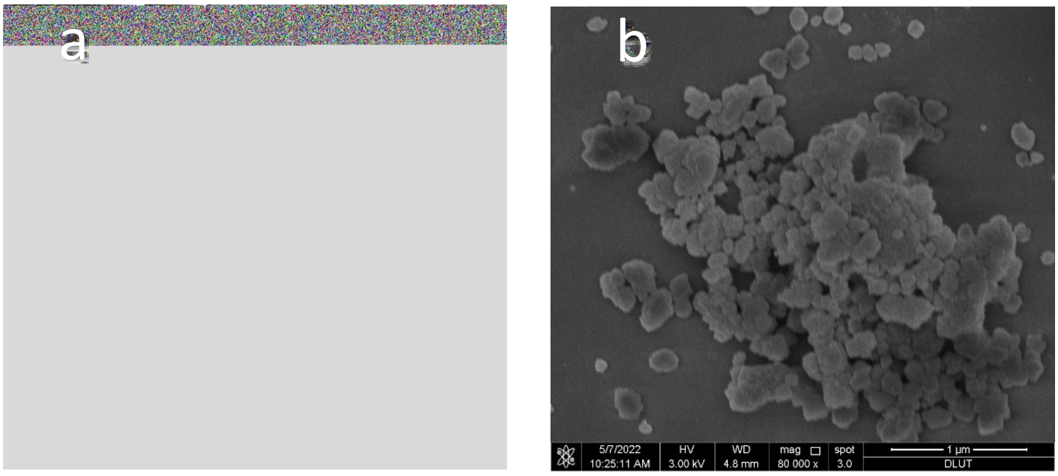

Fig. 6 SEM images of the Sn-Beta-2.5 (a), Sn-Beta-2.5-D (b)

Table 1

Textural properties of Sn-Beta-2.5 and Sn-Beta-2.5-D

Sample	S_{BET}[2)] m^2/g	S_{ext}[3)] m^2/g	V_{Total}[4)] cm^3/g	V_{micro}[5)] cm^3/g	V_{meso}[6)] cm^3/g	RC[7)] %
Sn-Beta-2.5-D	663	144	0.46	0.20	0.26	69
Sn-Beta-2.5	638	138	0.46	0.20	0.26	78

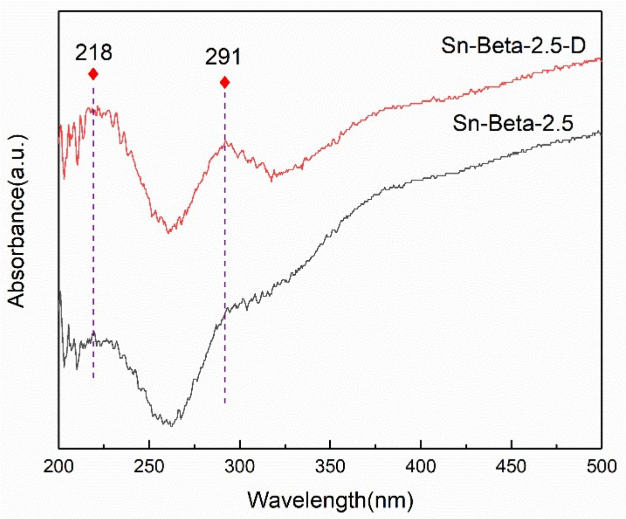

Fig. 7 UV-vis spectra of Sn-Beta-2.5 and Sn-Beta-2.5-D

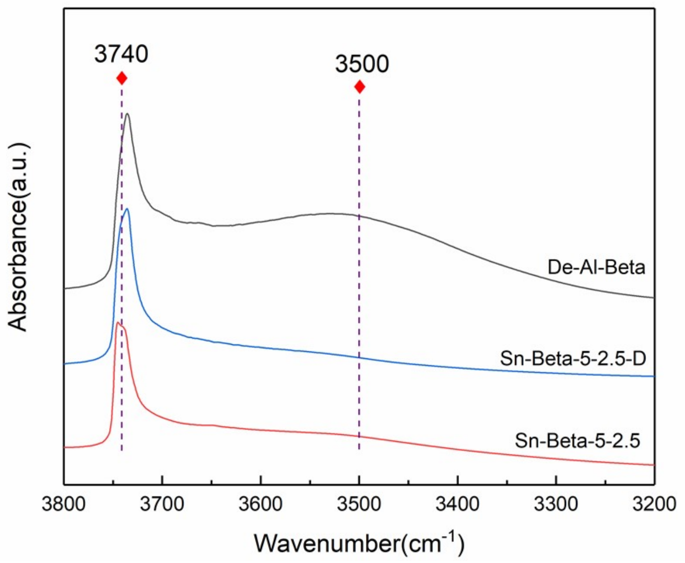

Fig.8 FT-IR spectra of the De-Al-Beta、Sn-Beta-2.5 and Sn-Beta-2.5-D

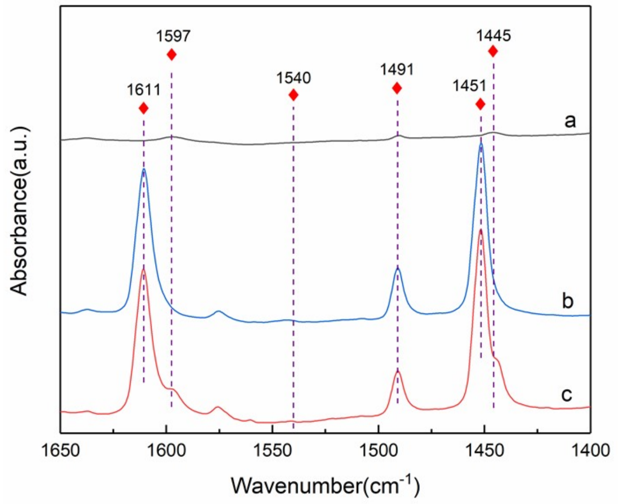

Fig. 9 FT-IR spectra of the De-Al-Beta、Sn-Beta-2.5 and Sn-Beta-2.5-D samples after pyridine adsorption at 298K for 30 min and desorption at 423K for 35 min

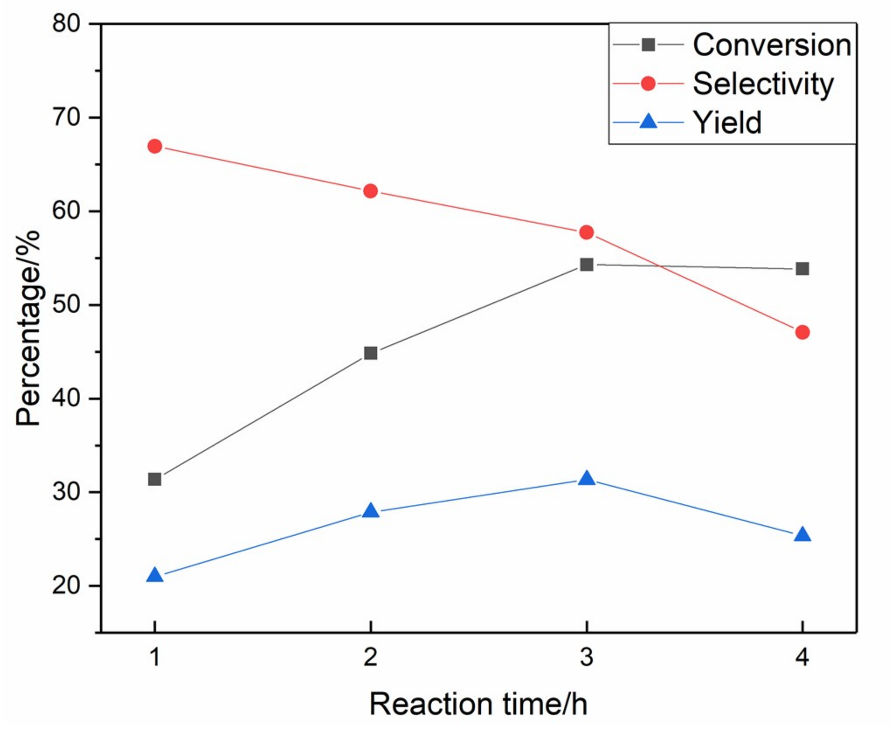

Fig. 10 Catalytic performances of the Sn-Beta-2.5 zeolite at different reaction times

Table 2

Table 5 Si/Sn molar ratios and Lewis acid contents of Sn-Beta-2.5-D and Sn-Beta-2.5

Sample	Si/Sn[1)]	LS[2)] μmol/g
Sn-Beta-5-2.5-D	33	144
Sn-Beta-5-2.5	37	171

注 [1)]Determined by ICP-OES;[2)]Calculating according to the equation given in the literature
